# Nonmedical Use of Cough Syrup Among Secondary Vocational School Students: A National Survey in China

**DOI:** 10.1097/MD.0000000000002969

**Published:** 2016-03-11

**Authors:** Qingfeng Wu, Jincong Yu, Chengwu Yang, Jiayan Chen, Longyu Yang, Hui Zhang, Shiwei Teng, Jiang Li, Dong Yan, Jiepin Cao, Yanting Zhao, Zengzhen Wang

**Affiliations:** From the Department of Epidemiology and Health Statistics, School of Public Health, Tongji Medical College, Huazhong University of Science and Technology, Wuhan (QW, JY, JC, LY, HZ, ST, DY, JC, YZ, ZW); Department of Preventive Medicine, Gannan Medical University, Ganzhou, China (QW); Department of Public Health Sciences & Office for Scholarship in Learning and Education Research, College of Medicine, Pennsylvania State University, Hershey, PA (CY); and Chongqing Health Information Center, Chongqing, China (JL).

## Abstract

Nonmedical use of cough syrup (NUCS) among secondary vocational school (SVS) students has been an increasing concern for public health in China, but no data were available. This cross-sectional study aimed to investigate the epidemiological characters of NUCS as well as its risk factors among SVS students in China.

From September 2013 to December 2014, a total of 13,614 SVS students were purposively selected through multistage sampling in 6 cities of China. Information on NUCS, demographics, family background, smoking and alcohol consumption, impulsiveness, sensation seeking, and parental monitoring were collected. Logistic regression was used to explore factors related to NUCS.

The 12,923 (94.9%) valid responses (16.3 ± 1.0 years old, and 52.6% men) reported 3.47% (95% confidence interval: 3.15–3.79%) lifetime NUCS. Logistic regression indicated that smoking, part-time job experience, high level of impulsiveness, and sensation seeking were risk factors for NUCS, whereas urban living and high parental monitoring were protective ones.

NUCS was prevalent among SVS students. Interventions that target on smoking, impulsiveness and sensation seeking control, improvement on parental monitoring may have considerable impact on NUCS among SVS students.

## INTRODUCTION

Cough syrup, especially for codeine-containing syrup (CCS), has been frequently used as an antitussive agent.^1^ Although its effects on the central nervous system are milder than heroin, the long-term use of CCS can lead to physical and psychological dependence.^2^ Nonmedical use of cough (NUCS) had become one of the commonly used abusive substances among adolescents in Western countries^3,4^ and some Asian countries.^5–9^ In China, cough syrup is commonly considered as addictive,^10–12^ given the 2 dominating types of cough syrups: 1 is codeine containing, the other is Chinese herbal cough syrup that contains components extracted from pericarpium papaveris, that is, poppy shell.^13^ There are very limited studies on the NUCS in China. A recent study in Guangdong province showed that the prevalence of nonmedical use of CCS was about 2.1% among regular high school (RHS) students,^14^ higher than illicit drug abuse in China (1%).^15^ However, currently there is no data on NUCS among secondary vocational school (SVS) students, a population at the same age of RHS students but usually are more prone to drug abuse and other social-behavioral problems in China.^16^ Since May, 2015, Chinese government had labeled CCS as category II psychoactive substances, which means that if 1 person traffics illegally would be punished as a drug dealer. Therefore, it is of great interest to investigate the epidemiological characteristics of NUCS among SVC students in China, and to know its prevalence and risk factors in order to gain better control over it.

It has been reported that NUCS is closely related with illicit drug abuse, brain damages, and psychological diseases.^9,17–20^ Studies suggested that NUCS might cause or co-exist with illicit drug use, and NUCS users are more inclined to become polydrug addicts.^19,20^ In addition, NUCS is also associated with damages to white matter of brain,^9^ the volume loss and aberrant functional organization in ventral medial prefrontal cortex,^21^ folate deficiency,^17,22^ and neural tube defects in fetus.^23^ Furthermore, the damages to brain in NUCS users may be linked to higher impulsivity,^9,21^ which in turn could lead to more serious drug-seeking behaviors.^18^ Hence, a vicious circle of drug misuse, brain damage, and higher impulsivity may be set up.

In China, SVS students are a special population with several features. First, mainly middle school graduates with poor academic performance will attend SVS, whereas others will attend RHS. Therefore, SVS students, owning to their poor academic performances at junior high schools, often feel inferior and lost.^24^ Second, problematic behaviors are common among SVS students, such as smoking, bullying, and addictive to internet or cell phone.^25^ Third, drug education among SVS students could not be effectively implemented.^26^ Based on these factors, we hypothesized that SVS students were more prone to develop substance abuse including NUCS. However, very few studies had focused on the problem among SVS students in China, and currently there are no epidemiological data of NUCS among them. This study aims: to assess the prevalence of NUCS among SVS students in China thorough a national survey, and to investigate factors (including demographics, problematic behaviors, and several psychological traits) that may be associated with NUCS in SVS students.

## METHODS

### Study Population

**FIGURE 1:**
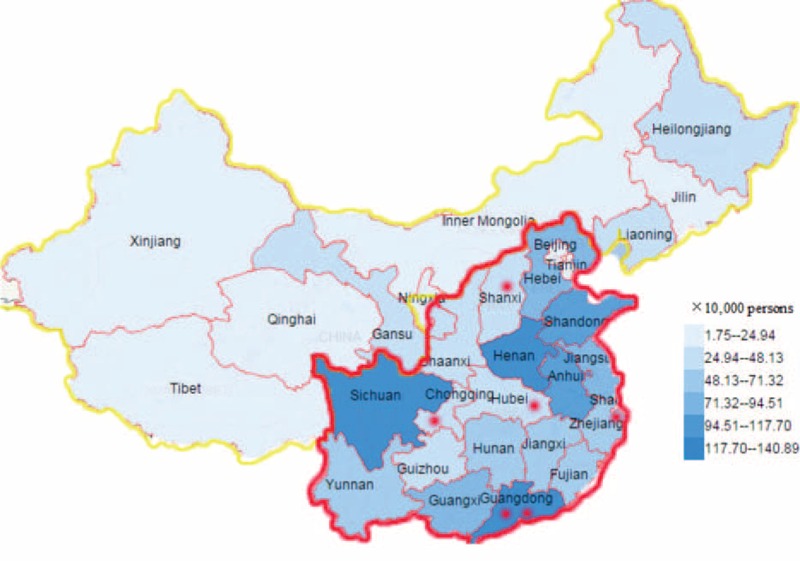
The distribution of secondary vocational (SVS) students and sampled areas. The mounts of SVS students in areas circled by a red line accounted for 84.9% of the nation. The red points marked the selected areas and cities where participants were sampled. Data from National Bureau of Statistics of China (http://data.stats.gov.cn). SVS = secondary vocational.

In our cross-sectional study, students at SVS in China were expected to enroll in the study. But data from National Bureau of Statistics of China showed that the amounts of SVS students in the marked region in Figure 1 were account for 84.9% (13.040 million out of 15.365 million) of the whole country in 2013.^27^ So students in the far-western region and northeast of China were not included because of small amounts of students. We adopted multistage clustering sampling strategies to select participants in the present study. In stage 1, the area in the red circle was divided into 5 blocks as North, South, East, West, and Center areas. Given Special Economic Zone (SEZ) as an extraordinary entity in Chinese economic construction, it was treated as the 6th block. Six cities were purposively sampled, including Shenzhen, Zhaoqing (South), Ningbo (East), Chongqing (West), Taiyuan (North), and Wuhan (Center) (as red points shown in Figure 1). In stage 2, given the variance of students number in each school, 2 or 3 schools were purposely sampled from each of the 6 selected cities, and 14SVSs were selected in total. In stage 3, students in year 1 and 2 were selected by cluster sampling from the 14 schools (students in grade 3 were not sampled because of graduation practice out of school). At last, 386 classes and a total of 14,195 students were sampled. However, 581 of them (4.1%) did not participate in the study because of illness or other reasons. This resulted in 13,614 participants, with 12,923 (94.9%) students provided valid information. The whole survey lasted from September 2013 to December 2014.

### Background Characters

Background information on participants’ demographics, academic performance, and monthly personal consumption expenditure were collected. The demographic variables included age, gender, race, habitual residence, and part-time job experience. Academic performance was assessed by average scores in the last semester and rated as 3 levels: (1) <60; (2) average (60–79); and (3) above average (80–100). The monthly expenditure was rated as the following categories: (1) <600 Yuan; (2) 600 to 999 Yuan; and (3) 1000 Yuan or more.

### Living Arrangement and Socio-economic Status (SES)

Information on living arrangement was collected by the question of “Who do you live with most of the time from childhood till now?” Living arrangement was classified as 3 types: (1) living with both parents (parents); (2) living with a single parent (single parent); and (3) living with other relatives (others).

SES is a comprehensive indicator being composed of parents’ education level and occupation. Because household income is difficult to collect, the item was omitted in the study. Information on parents’ education and occupation was ascertained through questionnaire. Education was rated as the following 3 levels: no more than junior high school, senior high school, and junior college and above. The 3 education levels were assigned the value 1, 2, and 3, respectively. Occupation was collected by 12 items including 11 close-ended items and 1 open-ended item. And according to the National Occupation Classification,^28^ occupations in this study were divided into the following 6 categories: unit leader (managerial), professionals, clerk, common merchant or service personnel, farmer, and other occupations difficult to categorize, which were assigned the value of 6, 5, 4, 3, 2, and 1, respectively. Finally, the family SES was assessed by summing the value of parents’ education and occupation according to a literature.^29^

### Smoking, Drinking, and NUCS

Smoking was assessed by asking “How many cigarettes a typical day do you smoke?” Daily cigarette consumption was divided into 4 levels: not at all, 1 to 9 cigarettes, 10 to 20 cigarettes, and >20 of cigarettes. Alcohol consumption was assessed by asking how many cups they had per drinking occasion. The alcohol in the study included beer, liquor, and wine. The unit of cup was used to measure alcohol consumption, and 1 cup defined as half bottle (250 mL) or 1 tin of beer, 25 mL distilled spirit, 100 mL wine, or 100 mL rice wine. The alcohol consumption was classified into 3 levels: not at all, 1 to 4 cups, and 5 cups or more.

NUCS was measured by the following question: How often did you use cough syrup without illness or just for “high” felling? The available choices for the frequency were never, ever tried, several times per month, several times per week, and every day. Lifetime NUCS was used as an indicator for judging users and nonusers. Respondents who had ever used cough syrup for nonmedical purpose or getting “high” feeling were defined as lifetime users.

### Impulsiveness and Sensation Seeking

Impulsiveness is 1 dimension in Substance Use Risk Profile Scale (SURPS). The SURPS was testified to be a good psychometric instrument.^30^ The subscale used in the study was developed by standard translation and back-translation procedures to ensure linguistic consistency. It was composed of 5 items, and the possible response on each item was scored from 1(strongly disagree) to 4 (strongly agree).

Sensation Seeking (SS) was measured by an 8-item subscale derived from the Form V of the Sensation Seeking Scale^31,32^ and was widely used due to its reliability.^33^ The Chinese version of Brief Sensation Seeking Scale (BSSS-C) was testified to be reliable and valid, and suitable for health risk behaviors prediction.^34^ A five-point Likert scale was used for scoring the BSSS-C ranged from 1 (completely disagree) to 5 (completely agree).

### Parental Monitoring (PM)

The scale of PM was extracted from the Communities That Care^®^ (CTC) Youth Survey instrument. The instrument had been originally described by Arthur et al,^35^ then some minor changes were made and developed into an 8-item scale.^36^ In the present study, 2 items (“If you carried a handgun without your parents’ permission, would you be caught by your parents?” and “Do your family has clear rules about alcohol and drug use?”) were excluded according to the Chinese cultural background and laws. Therefore, 6 of the 8 items were adopted and revised in this study. These 6 items were: “My parents ask if I’ve gotten my homework done,” “My parents would know if I did not come home on time,” “When I am out, one of my parents knows where I am and who I am with,” “If I skipped school would be punished by my parents,” “My family rules are very clear,” “If I drank beer or wine or liquor without my parents’ permission would be punished by my parents.” The option to each item was scored from 1(totally disagree) to 4 (totally agree).

### Statistical Methods

Data were double-entered and verified with EpiData version 3.1. Given participants came from different cities, the interclass correlation coefficient (*ICC*) was calculated among different cities to check whether multilevel model was conducted. However, results showed that the multilevel model was not suitable because of the low *ICC* (*ICC* = 0.037, *P* = 0.166). So Logistic regression analyses were conducted to explore factors related to NUCS. Quartile was used to divide quantitative variables into qualitative variables. All variables in the study were analyzed as dummy variables. Given the low proportion of missing data for all related variables (<1.1%), participants with missing data were not included in the nonconditional Logistic regression analysis. The statistical significance level was 2-sided at the 0.05 level. All statistical analyses were conducted with SAS version 9.4 (SAS Inc, Cary, NC).

### Ethnic Statement and Data Collection

At study design stage and before the study was conducted, Institutional Review Board (IRB) from the Medical Ethics Committee (MEC) of Tongji Medical College, Huazhong University of Science and Technology scrutinized and approved the study protocol, so that all ethical norms including the Helsinki norms were met. Given that all of the participating students are minor and that most of them are remote from their parents or guardians, we obtained agreement from the principals before the survey. Moreover, at the beginning of field survey, a well-trained investigator took several minutes to clearly elucidate the purpose and content of the survey to participants, the principles of confidentiality and voluntariness were emphasized to protect the participating students, and they could choose to refuse participating in the survey without any consequences. The students were all warranted that none of their parents, teachers, or peers would be aware of their responses. A total of 218 students refused to participate in the survey. During the survey, teachers were absent from the classroom. Data were collected during a single 40 minute classroom period by trained investigators following a standardized protocol.

## RESULTS

### Demographic characteristics of the participants

The mean age of the 12,923 individuals was 16.3 years old (±1.0 years), ranged from 13 to 20 years old. Among them, 52.6% were men, 58.6% were first grade students, 96.8% were Han Chinese, and 55.7% lived in urban areas in childhood. In their childhood, 76.7% of them lived with their parents and 22.9% of them lived with single parent or other relatives. As for academic achievement, 4161 (32.2%) students evaluated themselves as above average, and 6993 (54.1%) regarded themselves as average. In view of smoking and alcohol use, 79.9% of the students never smoked, and 43.9% never drank (Table 1).

**TABLE 1:**
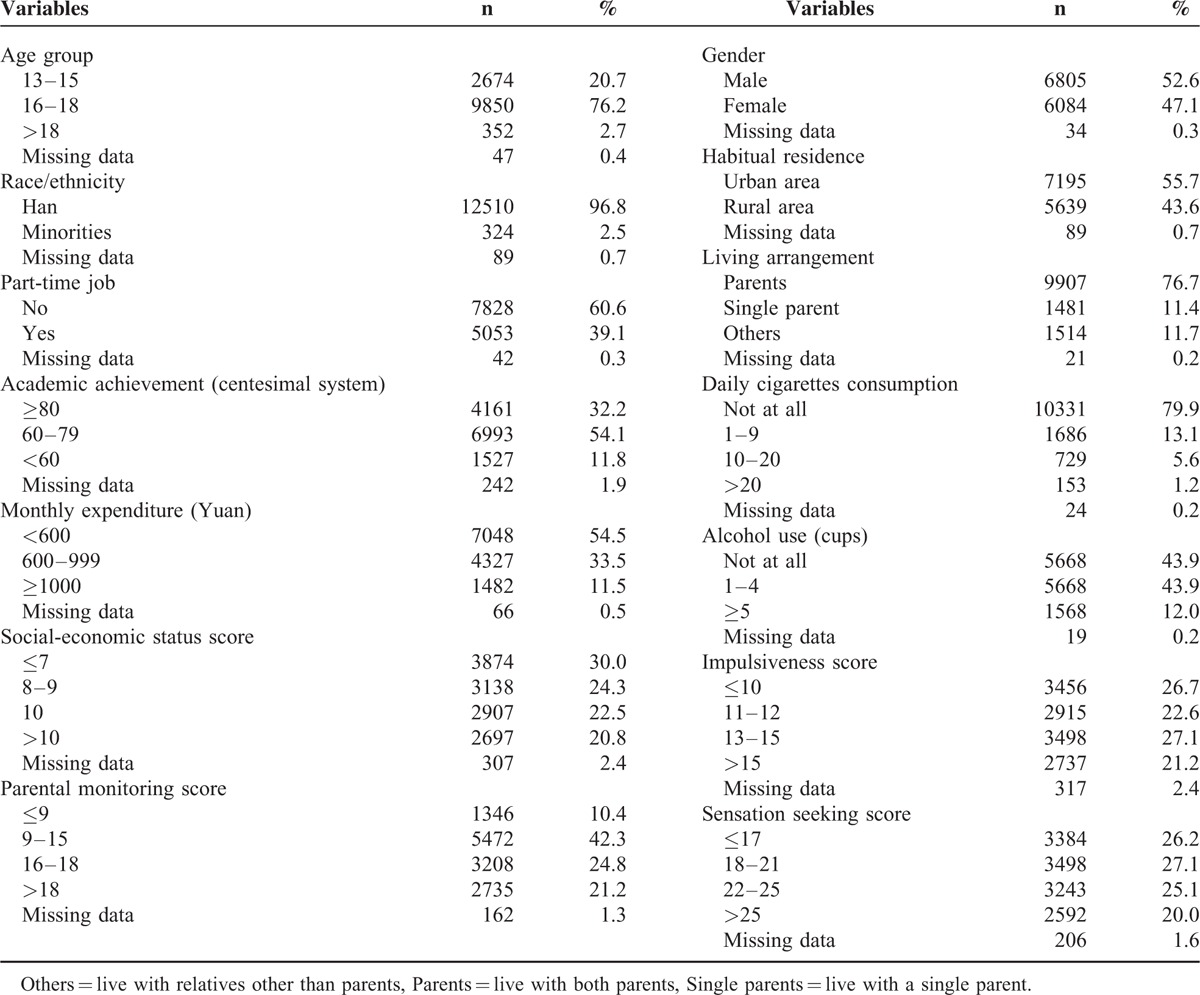
Demographic, Behavioral, and Psychological Characteristic of Secondary Vocational School Students

### Nonmedical use of cough syrup and its relationship with other factors

In the present study, the prevalence rate of lifetime NUCS was 3.47% (448 out of 12,923, 95% CI 3.15–3.79%). In order to explore the related factors associated with NUCS, univariate nonconditional Logistic regression analysis was carried out with NUCS as the dependent variable and other factors as independent variables. As Table 2 showed, without adjusting the influence of other variables, NUCS was more popular among students with characteristics of older age, male, living with people other than parents, having part-time job experiences, more monthly expenditure, more cigarettes and alcohol consumption, higher level of SS, and impulsiveness. However, students who had better academic achievements, lived in urban areas and families with high PM were less likely to use cough syrup nonmedically. No significant correlations were found between SES, ethnic, and NUCS in the present study.

**TABLE 2:**
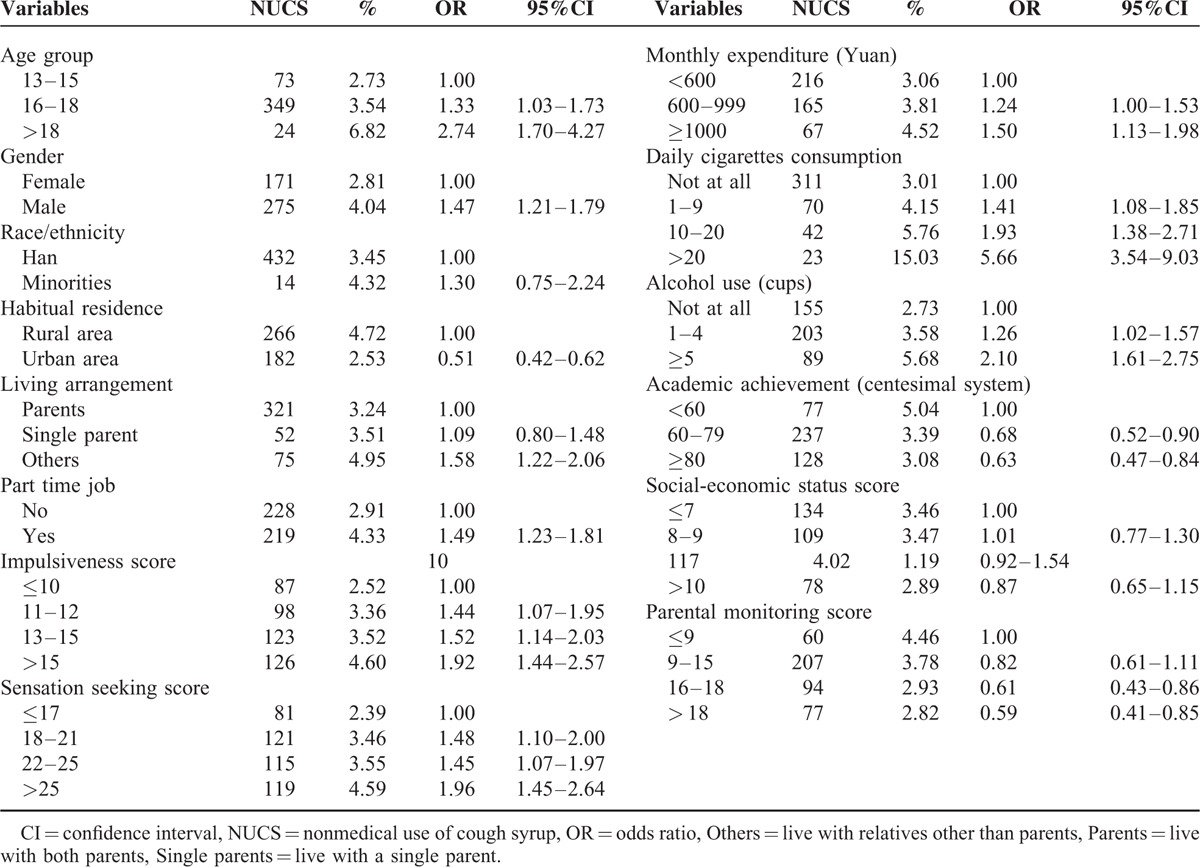
The Factors Related to Nonmedical Use of Cough Syrup by Univariate Nonconditional Logistic Regression Analysis

### Multivariate Logistic regression analysis on factors related to NUCS

All of the variables in Table 2 as potential predictors of NUCS were further analyzed by multivariate nonconditional Logistic regression model with stepwise selection method. Six variables retained in the final model: habitual residence, daily cigarette consumption, impulsiveness, SS, PM, and part-time job. Students living in urban areas had lower of NUCS than those living in rural areas. Smoking was positive related with NUCS, and the more cigarettes consumption, the higher risk of NUCS, especially for students who smoked >20 cigarettes per day. High level of sensation seeking and impulsiveness, taking part-time job experience were risk factors of NUCS. However, students with high level of PM were less likely to use cough syrup nonmedically (Table 3).

**TABLE 3:**
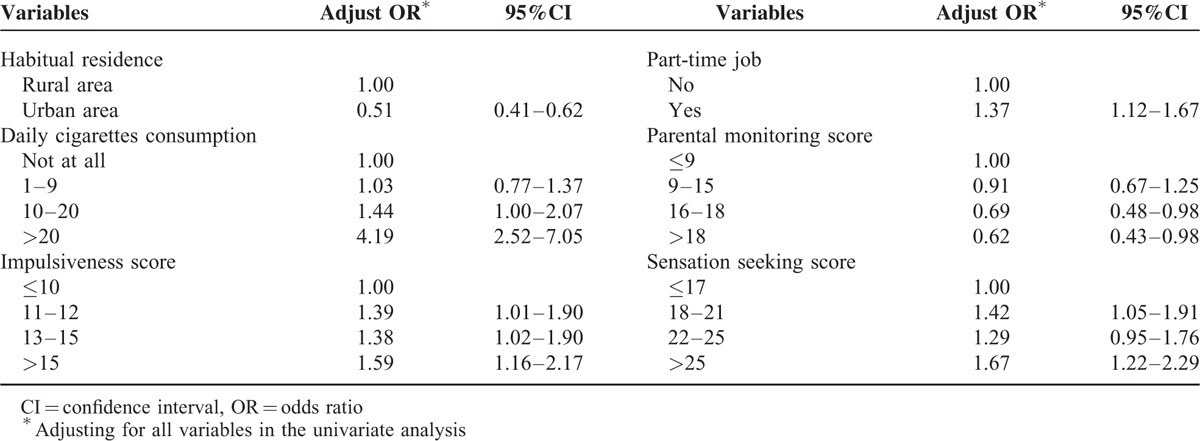
Factors Associated With Nonmedical Use of Cough Syrup by Multivariate Nonconditional Logistic Regression Analysis (n* *= 11,583)

## DISCUSSION

We found that the lifetime prevalence of NUCS was 3.47% in this study; and results also showed that more daily smoking consumption, high level of SS and impulsiveness, and part-time job experience could increase the risk of NUCS, whereas living in urban areas and high level of PM played a role in NUCS reduction.

The prevalence of NUCS varied across different counties and regions. The prevalence in our study was considerably lower than one study conducted among junior and high schools students in the United States in which the rate of the current cough syrup use was 9.5%.^37^ Two reasons may explain the difference. First, cough syrup has been popular among Chinese adolescents just for recent decades, so the prevalence may be lower. Second, psychoactive substance has been controlled rigorously by Chinese authority. Therefore, though 2 types of cough syrup were included in our study, the NUCS prevalence was still lower. However, the prevalence in our study was still higher than that of RHS students in Guangdong province, China, in which lifetime CCS misuse prevalence was 2.1%.^14^ Two aspects may account for the variance in the prevalence difference. First, our study included not only codeine containing cough syrup, but also some addictive Chinese herbal cough syrup. But in the preceding study in China, the cough syrup was confined within codeine containing cough syrup. Second, the study samples were also heterogeneous. The participants in our study were SVS students other than RHS students. As stated in Introduction, SVS students have more problematic behaviors^25^.

In our study, we found that more cigarettes consumption (>20 cigarettes per day, adjusted OR 4.2, 95% CI 2.52–7.05) was closely related to NUCS. Similar results were also reported in many other researches. Wang et al argured that smokers were more likely to report nonmedical use of prescription medicine than nonsmokers.^14^ Another study conducted among 85,000 adolescent students in 31 European countries also found that tobacco use was associated with nonmedical tranquilizer or sedative use (OR 1.3, 95%CI 1.1–1.5).^38^ Even among female students in grade 7 to 12, the close relationship was also found between smoking and nonmedical opioid use (OR 1.64, 95%CI 1.04–2.58).^39^ Therefore, interventions targeting adolescents’ smoking may still be effective in reducing prescription drug use, or in preventing the misuse of prescription and illicit drugs.

Experience of taking part-time jobs was found to be a risk factor and could increase the risk of NUCS in the present study. Several factors may account for the result. First, adolescents are deficit in knowledge on addictive drug,^40^ and most of them believe cough syrup is not addictive and harmless.^6^ Second, they have the characteristics of curiosity, emptiness, hopelessness, adventure, rebellion, and inferiority.^41^ At last, many SVS students often take part-time jobs in their spare time at factories, bars, hotels, travel agencies, and so on, in order to meet their career development. However, taking part-time jobs under the circumstances would increase the probability of exposure to NUCS and the risk to use this drug.

Notably, our research found that adolescents from rural areas had higher prevalence of NUCS than those of students from urban regions (4.7% vs 2.5%), similar results were reported by other studies. One study found that 34% of the rural RHS students reported nonmedical use of prescription drugs and was higher than that of the study reported nationally.^42^ The other national study conducted in 50 states in America showed that self-reported propoxyphene, codeine, and methadone misuse among rural residents were higher than their urban counterparts.^43^ Two possible reasons may be responsible for the situation in China. One is that prescription medicine management in rural areas is not as strict as urban places, and prescriptions might be easy to obtain.^44,45^ The other is that rural students know little about cough syrup, but when they went to cities and knew about it at schools, they tended to engage in overcompensation and develop NUCS behaviors.

Additionally, PM was found to be a protective factor of NUCS in our study. Namely, High level of PM is correlated with low NUCS. One study conducted among young participants aged 11 to 18 demonstrated that greater PM could reduce the likelihood of NUCS (OR 0.93, 95%CI 0.89–0.99).^46^ The similar effect of PM against adolescent substance use has also been demonstrated in several previous studies.^46–48^ Studies also found that parent–child bond along with PM would make adolescents feel warmth and love from their parents and be helpful in reducing substance misuse.^49–52^ Therefore, interventions targeting on improvement of PM and parent–child bond would be more effective for NUCS prevention than that of intervention targeting any one of them.

Many studies have testified that impulsivity and SS are correlated with prescription misuse, illicit drug abuse, and others problematic behaviors.^18,53–55^ In our study, we also found that SS and impulsiveness were closely related to NUCS among SVS students, and the risk of NUCS increased with the SS level ascent. Although SS is a stable personality determined mostly by biology genetic,^56^ 1 research had testified the validity of personality-targeted coping skills on substance misuse.^57^ Meanwhile, the relationship between SS and substance use may be mediated by impulsiveness, expectancies, and evaluation regarding nonmedical use of prescriptions and other substances abuse.^58–61^ Therefore, coping skills on SS and impulsiveness may be helpful to decrease the risk of NUCS.

### Limitations

Several limitations need to be considered when evaluating the study's findings. First, given the study is cross-sectional, caution should be warranted in making causal interpretations. Longitudinal studies are necessary to clarify the relationships of these factors. Second, the nonprobability sampling strategies may affect the generalization of the result. Third, some cases were excluded from the study because of invalidity. But the difference of basic information and NUCS between valid and invalid participants did not meet the statistical significance.

## CONCLUSION

In sum, this study demonstrates that NUCS is prevalent among SVS students in China. Health education associated with psychoactive elements containing prescriptions, smoking, and narcotics should be reinforced among the population. Skills on SS and impulsiveness control may be helpful to reduce drug seeking behaviors. Given PM is negatively related with NUCS, improvement on PM especially for families in rural regions could contribute to preventing NUCS among SVS students.

## References

[1] Hutchings HA Eccles R The opioid agonist codeine and antagonist naltrexone do not affect voluntary suppression of capsaicin induced cough in healthy subjects. *Eur Respir J* 1994; 7:715–719. 8005254 10.1183/09031936.94.07040715

[2] Vree TB van Dongen RT Koopman-Kimenai PM Codeine analgesia is due to codeine-6-glucuronide, not morphine. *Int J Clin Pract* 2000; 54:395–398. 11092114

[3] Agnich LE Stogner JM Miller BL Purple drank prevalence and characteristics of misusers of codeine cough syrup mixtures. *Addict Behav* 2013; 38:2445–2449. 23688907 10.1016/j.addbeh.2013.03.020

[4] Martins SS Keyes KM Storr CL Birth-cohort trends in lifetime and past-year prescription opioid-use disorder resulting from nonmedical use: results from two national surveys. *J Stud Alcohol Drugs* 2010; 71:480–487. 20553656 10.15288/jsad.2010.71.480 PMC2887918

[5] Mattoo SK Basu D Sharma A Abuse of codeine-containing cough syrups: a report from India. *Addiction* 1997; 92:1783–1787. 9581010

[6] Shek DTL Lam CM Beliefs about cough medicine abuse among Chinese young people in Hong Kong. *Soc Behav Pers* 2008; 36:135–144.

[7] Ishigooka J Yoshida Y Murasaki M Abuse of “BRON”: a Japanese OTC cough suppressant solution containing methylephedrine, codeine, caffeine and chlorpheniramine. *Prog Neuropsychopharmacol Biol Psychiatry* 1991; 15:513–521. 1749828 10.1016/0278-5846(91)90026-w

[8] Shek DT Lam CM Adolescent cough medicine abuse in Hong Kong: implications for the design of positive youth development programs in Hong Kong. *Int J Adolesc Med Health* 2006; 18:493–503. 17068932 10.1515/ijamh.2006.18.3.493

[9] Qiu YW Su HH Lv XF Abnormal white matter integrity in chronic users of codeine-containing cough syrups: a tract-based spatial statistics study. *Am J Neuroradiol* 2015; 36:50–56. 25104290 10.3174/ajnr.A4070 PMC7965918

[10] China Food Drug Administration, Xu J Four Strict Measures on Cough Syrup Abuse in Shenzhen. 2013; 56.

[11] Zhang C He X The awkward of cough syrup. *China Health Industry* 2008; 5:96.

[12] Fu Z Why cough syrup is abused. People's Daily. 21 May 2010; pp 009.

[13] Modern Hospital, He R Xiao X The Condition and Countermeasures on Cough Mixture Addition. 2006; 32–34.

[14] Wang H Deng J Zhou X The nonmedical use of prescription medicines among high school students: a cross-sectional study in Southern China. *Drug Alcohol Depend* 2014; 141:9–15. 24875678 10.1016/j.drugalcdep.2014.04.004

[15] The State Council Information Office of the People's Republic of China. Press Conference of China Drug Report 2014. June 2015 Available at http://www.scio.gov.cn/xwfbh/xwbfbh/wqfbh/2015/32959/index.htm Assessed on 6 September, 2015.

[16] LI J The review of risk behaviors among secondary occupational school students in China. *J Changchun Educ Inst* 2014; 30:138–139.

[17] Au W-y Harod KK Law M-f Cough mixture abuse, folate deficiency and acute lymphoblastic leukemia. *Leukemia Res* 2009; 33:508–509. 18715641 10.1016/j.leukres.2008.07.007

[18] Dennhardt AA Murphy JG Prevention and treatment of college student drug use: a review of the literature. *Addict Behav* 2013; 38:2607–2618. 23846178 10.1016/j.addbeh.2013.06.006

[19] Jones CM Heroin use and heroin use risk behaviors among nonmedical users of prescription opioid pain relievers—United States, 2002–2004 and 2008–2010. *Drug Alcohol Depend* 2013; 132:95–100. 23410617 10.1016/j.drugalcdep.2013.01.007

[20] McCabe SE Boyd CJ Young A Medical and nonmedical use of prescription drugs among secondary school students. *J Adolesc Health* 2007; 40:76–83. 17185209 10.1016/j.jadohealth.2006.07.016 PMC1764616

[21] Qiu YW Lv XF Jiang GH Reduced ventral medial prefrontal cortex (vmPFC) volume and impaired vmPFC-default mode network integration in codeine-containing cough syrups users. *Drug Alcohol Depend* 2014; 134:314–321. 24286968 10.1016/j.drugalcdep.2013.10.023

[22] Au WY Tsang SK Cheung BK Cough mixture abuse as a novel cause of folate deficiency: a prospective, community-based, controlled study. *Haematologica* 2007; 92:562–563. 17488670 10.3324/haematol.10859

[23] Au WY Cheng TS Siu TS Cerebellar degeneration and folate deficiency due to cough mixture abuse. *Haematologica* 2005; (90 Suppl):ECR28–ECR128. 16266919

[24] Jia F Guo L Feng C A study of the mental health of secondary vocational students in Suzhou and educational Strategies. *J Suzhou Sci Technol (Nat Sci)* 2010; 27:77–80.

[25] Zhang Z Li L The comparison of health risk behaviors between students in secondary vocational school and senior high school. *Chin J Sch Health* 2013; 34:1124–1125.

[26] Su X Zheng L Tian H Survey about the knowledge on drugs in the nursing students in Handan vocational schools. *Mod Prev Med* 2010; 37:2856–2857.

[27] National Bureau of Statistics of China. Number of Enrollment in Undergraduate Courses of Secondary Vocational School in 2013. Available at http://data.stats.gov.cn/english/mapdata.htm?cn=E0103&zb=A0301 Assessed on 28 January 2016.

[28] National Occupational Classification and Vocational Qualification Authentication Committee. *National Occupational Classification of the People's Republic of China*. Beijing:China Labor Press; 1999.

[29] Shi B Shen J The relationships among family SES, intelligence, intrinsic motivation and creativity. *Psychol Dev Educ* 2007; 23:30–34.

[30] Woicik PA Stewart SH Pihl RO The Substance Use Risk Profile Scale: a scale measuring traits linked to reinforcement-specific substance use profiles. *Addic Behav* 2009; 34:1042–1055. 10.1016/j.addbeh.2009.07.001 19683400

[31] Hoyle RH Stephenson MT Palmgreen P Reliability and validity of a brief measure of sensation seeking. *Pers Individual Differences* 2002; 32:401–414.

[32] Zuckerman M Eysenck S Eysenck HJ Sensation seeking in England and America: cross-cultural, age, and sex comparisons. *J Consult Clin Psychol* 1978; 46:139–149. 627648 10.1037//0022-006x.46.1.139

[33] Vallone D Allen JA Clayton RR How reliable and valid is the Brief Sensation Seeking Scale (BSSS-4) for youth of various racial/ethnic groups? *Addiction* 2007; 102 (Suppl 2):71–78. 17850616 10.1111/j.1360-0443.2007.01957.x

[34] Chen X Li F Nydegger L Brief sensation seeking scale for Chinese-cultural adaptation and psychometric assessment. *Pers Individ Dif* 2013; 54:604–609. 23316097 10.1016/j.paid.2012.11.007 PMC3539791

[35] Arthur MW Hawkins JD Pollard JA Measuring risk and protective factors for substance use, delinquency, and other adolescent problem behaviors. The Communities That Care Youth Survey. *Eval Rev* 2002; 26:575–601. 12465571 10.1177/0193841X0202600601

[36] Glaser RR Horn MLV Arthur MW Measurement properties of the Communities That Care® Youth Survey across demographic groups. *J Quant Criminol* 2005; 21:73–102.

[37] Peters RJ Jr Kelder SH Markham CM Beliefs and social norms about codeine and promethazine hydrochloride cough syrup (CPHCS) onset and perceived addiction among urban Houstonian adolescents: an addiction trend in the city of lean. *J Drug Educ* 2003; 33:415–425. 15237866 10.2190/NXJ6-U60J-XTY0-09MP

[38] Kokkevi A Fotiou A Arapaki A Prevalence, patterns, and correlates of tranquilizer and sedative use among European adolescents. *J Adolesc Health* 2008; 43:584–592. 19027647 10.1016/j.jadohealth.2008.05.001

[39] Brands B Paglia-Boak A Sproule BA Nonmedical use of opioid analgesics among Ontario students. *Can Fam Physician* 2010; 56:256–262. 20228312 PMC2837694

[40] Yang X Xia G Causes and consequences of increasing club drug use in China: a descriptive assessment. *Subst Use Misuse* 2010; 45:224–239. 20025450 10.3109/10826080903039827

[41] Madruga CS Laranjeira R Caetano R Use of licit and illicit substances among adolescents in Brazil—a national survey. *Addict Behav* 2012; 37:1171–1175. 22703876 10.1016/j.addbeh.2012.05.008

[42] Levine SB Coupey SM Nonmedical use of prescription medications: an emerging risk behavior among rural adolescents. *J Adolesc Health* 2009; 44:407–409. 19306802 10.1016/j.jadohealth.2008.08.010

[43] Wang KH Becker WC Fiellin DA Prevalence and correlates for nonmedical use of prescription opioids among urban and rural residents. *Drug Alcohol Depend* 2013; 127:156–162. 22819293 10.1016/j.drugalcdep.2012.06.027

[44] Wang L Chen H Yu S Surveying the management status of rural involved institution's drug storage. *Chin Health Serv Manag* 2010; 27:454–455.

[45] Sun J Current situation and countermeasures on regulation of prescription drugs sale of pharmaceutical retail enterprises without prescription in China. *Qilu Pharm Aff* 2006; 25:275–276.

[46] Cheng TC Lo CC Nonmedical use of prescription medications: a longitudinal analysis with adolescents involved in child welfare. *Child Youth Serv Rev* 2012; 34:859–864.

[47] Ford JA Nonmedical prescription drug use among adolescents: the influence of bonds to family and school. *Youth Soc* 2008; 40:336–352.

[48] Ledoux S Miller P Choquet M Family structure, parent–child relationships, and alcohol and other drug use among teenagers in France and the United Kingdom. *Alcohol Alcohol* 2002; 37:52–60. 11825858 10.1093/alcalc/37.1.52

[49] Donaldson CD Nakawaki B Crano WD Variations in parental monitoring and predictions of adolescent prescription opioid and stimulant misuse. *Addict Behav* 2015; 45:14–21. 25622102 10.1016/j.addbeh.2015.01.022 PMC5902021

[50] Cheng TC Lo CC A longitudinal analysis of some risk and protective factors in marijuana use by adolescents receiving child welfare services. *Child Youth Serv Rev* 2011; 33:1667–1672.

[51] Tucker JS Ellickson PL Collins RL Are drug experimenters better adjusted than abstainers and users? a longitudinal study of adolescent marijuana use. *J Adolesc Health* 2006; 39:488–494. 16982382 10.1016/j.jadohealth.2006.03.012

[52] Hoffmann JP Cerbone FG Parental substance use disorder and the risk of adolescent drug abuse: an event history analysis. *Drug Alcohol Depend* 2002; 66:255–264. 12062460 10.1016/s0376-8716(02)00005-4

[53] Herman-Stahl MA Krebs CP Kroutil LA Risk and protective factors for methamphetamine use and nonmedical use of prescription stimulants among young adults aged 18 to 25. *Addict Behav* 2007; 32:1003–1015. 16920275 10.1016/j.addbeh.2006.07.010

[54] Low KG Gendaszek AE Illicit use of psychostimulants among college students: a preliminary study. *Psychol Health Med* 2002; 7:283–287.

[55] Arria AM Caldeira KM Vincent KB Perceived harmfulness predicts nonmedical use of prescription drugs among college students: interactions with sensation-seeking. *Prev Sci* 2008; 9:191–201. 18633709 10.1007/s11121-008-0095-8 PMC2574828

[56] Xu J Yang L The correlation between sensation seeking and long latency auditory evoked potentials. *Chin J Behav Med Sci* 2002; 11:708–709.

[57] Conrod PJ Castellanos-Ryan N Strang J Brief, personality-targeted coping skills interventions and survival as a non-drug user over a 2-year period during adolescence. *Arch Gen Psychiatry* 2010; 67:85–93. 20048226 10.1001/archgenpsychiatry.2009.173

[58] Acton GS Measurement of impulsivity in a hierarchical model of personality traits: implications for substance use. *Subst Use Misuse* 2003; 38:67–83. 12602807 10.1081/ja-120016566

[59] Lookatch SJ Dunne EM Katz EC Predictors of nonmedical use of prescription stimulants. *J Psychoactive Drugs* 2012; 44:86–91. 22641970 10.1080/02791072.2012.662083

[60] Steele CM Josephs RA Alcohol myopia. Its prized and dangerous effects. *Am Psychol* 1990; 45:921–933. 2221564 10.1037//0003-066x.45.8.921

[61] Zuckerman M Behavioral Expressions and Biosocial Bases of Sensation Seeking. 1994; Cambridge:Cambridge University Press, pp. 225–257.

